# Hormonal interventions in skin wounds – a mini review

**DOI:** 10.1186/s10020-024-00978-6

**Published:** 2024-11-14

**Authors:** Zeming Li, Rui Ma, Jiajun Tan, Chunmeng Li, Yang Xiao, Xudong Qiu, Shuo Jin, Peng Ouyang, Yiping Zhao, Xiao Xiang, Wang Wu

**Affiliations:** 1 College of Bioengineering, Chongqing University, Chongqing, 400044 China; 2 Institute of Burn Research, Southwest Hospital, State Key Lab of Trauma, Burn and Combined Injury, Chongqing Key Laboratory for Disease Proteomics, Third Military Medical University, Chongqing, 400038 China; 3 Chongqing Yi-Cheng Biotechnology Co., LTD, Chongqing, 400004 China; 4 Three Gorges Hospital of Chongqing University, Chongqing, 400004 China; 5 China Medical University, Shenyang, 110001 China; 6 Department of Dermatology, The First Hospital of China Medical University, Shenyang, 110001 China; 7 Key Laboratory of Immunodermatology, Ministry of Education and NHC, National joint Engineering Research Center for Theranostics of Immunological Skin Diseases, Shenyang, 110001 China; 8 Department of General Surgery, The First Affiliated Hospital of Jinan University, Guangzhou, 510632 China; 9 The Affiliated Hospital of Beihua University, Jilin, 224000 China

**Keywords:** Wound healing, Hormone, Growth factors, Hormonal interventions

## Abstract

The ability to heal from wounds is perhaps the most important biological function that ensures our survival and perpetuation. Cutaneous wound healing typically consists of four characteristic stages, namely hemostasis, inflammation, proliferation, and remodeling, which are carefully carried out by coordinated actions of various cells, cytokines, and hormones. Incoordination of these steps may impede complete and efficient reconstruction and functional recovery of wounds or even lead to worsened outcomes. Hormones, as powerful modulators of organ functions, participate in multiple steps of the wound healing process and play a pivotal role by choreographing the complex interplay of cellular and molecular events. Leveraging the regulatory effects of hormones to enhance the healing process, hormonal therapy has emerged as a promising approach in the clinical treatment of wounds. Current research has focused on determination of the optimal dosages, delivery methods, and combinations of hormonal therapies to maximize their therapeutic benefits while minimizing potential side effects. This review highlights the molecular mechanisms, clinical benefits and side effects of the most commonly used hormones in clinical treatment of wounds.

## Introduction

**Fig. 1:**
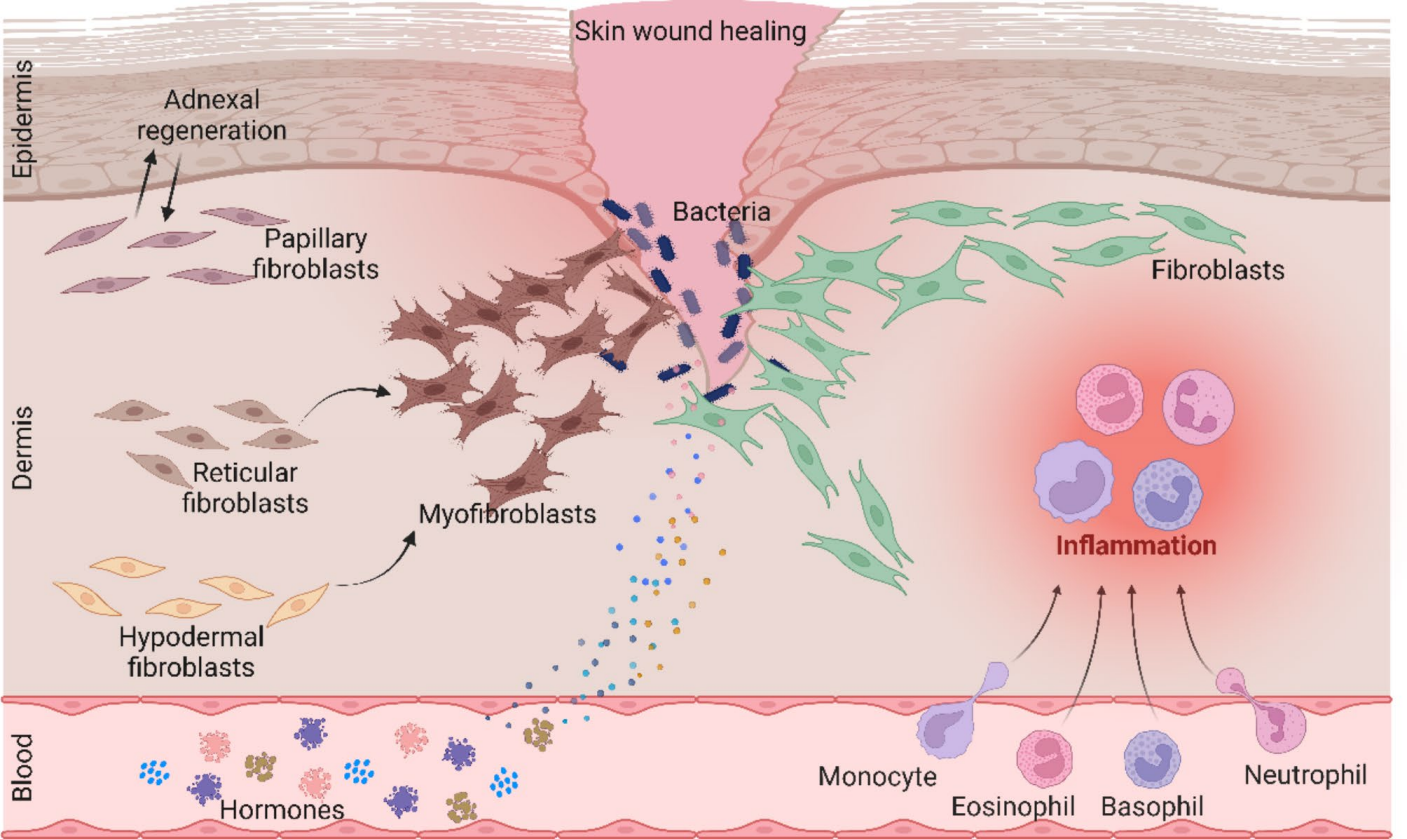
The process of skin wound healing. Wound healing is a sophisticated biological process that involves the orchestrated interaction of various tissues, cells, and factors. It encompasses cellular migration, proliferation, extracellular matrix synthesis and remodeling, as well as the precise regulation of inflammatory responses and angiogenesis

Skin wound healing is a complex biological process, consisting of inflammatory, hyperplastic, and tissue remodeling stages, involving various cell types, growth factors, and hormones (Peña et al. 2024) (Fig. 1). In the early stages of wound healing, inflammatory cells such as neutrophils, monocytes, eosinophils, and basophils respond rapidly to the injury and migrate to the wound site to remove necrotic tissue and pathogens, thus creating a favorable environment for tissue reconstruction to take place (LeBert et al. 2014; Lopes et al. 2024). At the same time, many growth factors and hormones are released to the wound through blood circulation (Fig. 1) to initiate and aid the healing process. After formation of the basal layer of the epidermis, fibroblasts from the underlying papillary layer migrate, attach, and aggregate to form dermal clots (Frech et al. 2022). The interaction of dermal coagulation and epidermal substrate allows skin appendages such as hair follicles at the edge of the wound to grow into the dermis (Woodley 2017). In the dermal layer of the wound, the reticular fibroblasts secrete a large amount of extracellular matrix (ECM) and migrate to the wound, forming granulation tissue together with immune cells. The dermal fibroblasts are then subjected to mechanical stress from the ECM, which induces the production of transforming growth factor beta 1 (TGF-β1), that promotes the transformation of dermal fibroblasts into myofibroblasts (Fig. 1), allowing the wound to contract and close (Boraldi et al. 2024). In parallel, epidermal cells gradually migrate to the middle of the wound to re-epithelialize the wound via rapid proliferation and differentiation. In the final stages of wound healing, active myofibroblasts are transformed into mature fibroblasts, leading to complete wound healing (Rippa et al. 2019; Jin et al. 2023).

In the recent years, the application of hormones in the clinical management of skin wounds has garnered significant attention due to their profound influence on various phases of wound healing (Wen et al. 2022; Wang et al. 2024). In clinical practice, several hormones have been utilized to manage skin wounds, including recombinant human growth hormone (RHGH), which has been shown to enhance tissue regeneration and control inflammation (Breederveld et al. 2012; Breederveld et al. 2014). Estrogen has also been implicated in promoting angiogenesis and improving collagen deposition (Horng et al. 2017). Androgens are known to have similar effects (Huang et al. 2024). Additionally, glucocorticoids are widely used for their anti-inflammatory properties and role in wound healing, although they require careful administration due to potential side effects (Brazel et al. 2020). These hormonal interventions, tailored to the specific needs and hormonal milieu of patients, represent a promising adjunct to conventional wound care therapies, aiming to optimize the healing process and reduce the incidence of chronic wounds. However, systemic effects of hormones on wound healing are mainly judged by laboratory tests and the potential side effects of various hormones on wound healing are still not fully understood. In view of this, this review aimed to summarize the effects of various hormones on skin wound healing under clinical settings, elucidate their mechanisms of action, assess their potential clinical applications, and critically evaluate the associated side effects to provide a comprehensive understanding of their utility in wound management.

## Recombinant human growth hormone

Recombinant human growth hormone (RHGH), produced through gene recombination technology, accelerates the healing process by facilitating collagen deposition and increasing reserves of essential elements like nitrogen, phosphorus, and potassium at the wound site (Herndon et al. 1995). Additionally, RHGH promotes protein synthesis and improves muscle protein metabolism, contributing to better overall treatment outcome (Ren et al. 2022). Clinical benefits of RHGH for skin wound treatment, especially for burns, include stimulating the liver to produce insulin-like growth factor-1 (IGF-1), which enhances the healing rate of donor sites for skin grafts and reduces healing time. This acceleration of healing is crucial for minimizing the duration of hospitalization and lowering infection risks (Herndon et al. 1995). Furthermore, clinical trials indicate that the use of RHGH does not increase scar formation, thus offering a new therapeutic strategy for scar management post-injury (Barret et al. 1999). In the treatment of pressure ulcers, the local application of RHGH also shows a promoting effect on healing, not only accelerating the healing rate but also improving the quality of healing. This improvement is evident by the increased skin thickness in the healing area and the improved Collagen I/II ratio, which helps reduce the recurrence of pressure ulcers and enhance the patients’ quality of life (Cristóbal et al. 2019).

Some studies have used animal models to study the mechanism of RHGH in wound healing. It has been reported that local application of RHGH accelerates the healing rate of pressure ulcers in a human skin mouse model, showing a significantly faster healing rate in the treated group compared to the control group, particularly during the initial 30 days post-treatment (Cristóbal et al. 2019). This rapid response to RHGH treatment not only suggests a potential for improved healing times but also indicates that RHGH may contribute to the structural integrity of the regenerated skin, as evidenced by the thicker skin and the significant reduction in the collagen type I/III ratio at the time of wound closure. Further research has extended these findings to more severe skin traumas, such as burns (Shao et al. 2024). RHGH has been found to enhance wound healing capacity and alleviate inflammatory and oxidative stress responses in burned mice. The underlying mechanism appears to involve the activation of the extracellular signal-regulated kinase (ERK) pathway, as indicated by increased expression of proteins like p-ERK1/2/ERK1/2, epidermal growth factor (EGF), TGF-β, and vascular endothelial growth factor (VEGF) (Shao et al. 2024). This suggests that RHGH could be crucial in the management of skin injuries that require a more complex healing process, highlighting its potential in treating severe skin conditions beyond simple wounds.

### Estrogen

Estrogen plays a pivotal role in the clinical treatment of skin wounds due to its multifaceted effects on the wound healing process. It has been demonstrated that estrogen can significantly enhance the various stages of wound healing of both genders, including inflammation, proliferation, and remodeling (Samantaray et al. 2016a; Shvetcov et al. 2023). Estrogen exerts anti-inflammatory effects by modulating cytokine production and reducing the infiltration of neutrophils and macrophages, which helps in controlling excessive inflammation and preventing chronic wound formation (Mukai et al. 2022). Additionally, estrogen promotes re-epithelialization by enhancing keratinocyte migration and proliferation (Farzan et al. 2014; Wang et al. 2022). Estrogen also stimulates angiogenesis, the formation of new blood vessels, thereby improving blood supply and nutrient delivery to the wound site (Bernelot Moens et al. 2012; Samantaray et al. 2016). Moreover, estrogen enhances collagen synthesis and deposition, leading to stronger and more resilient tissue repair (Ahn et al. 2020). Clinical studies have shown that estrogen therapy, either systemic or topical, can accelerate wound healing and improve the quality of the healed tissue, particularly in postmenopausal women who often experience delayed wound healing due to decreased estrogen levels (El Mohtadi et al. 2021). Thus, estrogen represents a valuable therapeutic agent in enhancing wound healing, particularly in populations with compromised estrogen levels.

Mechanistically, estrogen binds directly to specific receptors in the skin, particularly estrogen receptor α (ERα) and estrogen receptor β (ERβ), which are expressed in keratinocytes and activated upon estrogen binding (Moeinpour et al. 2008). This binding and activation are critical steps in estrogen’s promotion of skin repair (Bernelot Moens et al. 2012; Samantaray et al. 2016a). Following receptor binding, estrogen activates a series of signaling pathways, including the ERK1/2 signaling pathway, which is crucial for the proliferation and differentiation of keratinocytes. Specifically, the activation of the ERK1/2 pathway advances the cell cycle, thereby increasing cell numbers (Yan et al. 2011). Estrogen also enhances the activation of growth factor receptors, such as the transmembrane tyrosine kinase growth factor receptor, indirectly promoting the proliferation of keratinocytes, thus enhancing the regenerative capacity of skin (Yan et al. 2011). Moreover, estrogen can also indirectly influence skin wound healing by stimulating macrophages to produce nerve growth factor (NGF) and VEGF (Wang et al. 2022). These factors promote the proliferation and migration of keratinocytes, thereby accelerating wound healing (Rujirachotiwat et al. 2021; Wang et al. 2022). NGF and VEGF not only enhance keratinocyte proliferation but also increase nerve and epidermis regeneration at the wound site, as well as granulation tissue formation (M, et al. 2023; Sych et al. 2023).

## Androgens

Androgens, such as testosterone, have emerged as prominent agents in the clinical treatment of skin wounds, particularly due to their anabolic effects on tissue repair and regeneration (Bentley et al. 2023). Evidence so far suggests that androgens facilitate the wound healing process by enhancing the proliferation and differentiation of keratinocytes and fibroblasts, which are essential for re-epithelialization and collagen deposition (Cousins et al. 2016). Aside from promoting angiogenesis during wound healing, androgens exert immunomodulatory effects, such as reducing pro-inflammatory cytokines at the wound, which is critical in preventing chronic wounds and promoting efficient healing (Chi et al. 2024).

In spite of its therapeutic effects, recent studies however indicate that, in certain situations, androgens may inhibit skin wound healing. For instance, in conditions of chronic inflammation or when there is an overproduction of inflammatory cytokines, androgens may exacerbate the inflammatory response (Shi et al. 2021). Specifically, androgens have been shown to increase the production of interleukin-6 (IL-6) and tumor necrosis factor-alpha (TNF-α), which can lead to a prolonged inflammatory phase and hinder the wound healing process (Shi et al. 2021). This effect is particularly relevant in cases such as diabetic wounds and major burn wound healing, where the inflammatory response is often more pronounced and prolonged. The prolongation of the inflammatory response not only slows down the wound healing process but may also cause further damage to the surrounding tissues (Gärtner et al. 2021; Shi et al. 2021). This includes reducing epithelial formation and extracellular matrix deposition in the wound area, which directly affects cell proliferation and collagen synthesis required for wound healing (Gärtner et al. 2021). Additionally, it was indicated that the inhibition of wound healing by androgens might be exacerbated by stress (Romana-Souza et al. 2014). Although some studies suggest that testosterone or oxandrolone can improve weight maintenance, increase muscle protein metabolism, and shorten hospital stays (Shankaran et al. 2016), the use of androgens may require particular caution in high-stress situations such as severe trauma, the recovery period after major surgery, or cachexia caused by chronic diseases (Hundeshagen et al. 2024). In these contexts, androgens may have inhibitory effects on wound healing, which could counteract their anabolic benefits (Shi et al. 2020; Huang et al. 2024). So, the dual effects of androgen therapy on muscle synthesis and wound healing must be carefully weighed, especially in high-risk populations where the balance between these outcomes is critical. To mitigate the systemic side effects of androgens, researchers have developed novel electro-spun polycaprolactone (PCL) scaffold wound dressings for localized, sustained delivery of anti-androgens. Additionally, a synthetic androgen, dihydrotestosterone (DHT), promotes local wound healing by reducing the infiltration of macrophages into the wound (Shi et al. 2020).

## Glucocorticoids

Glucocorticoids are steroid hormones secreted by the adrenal cortex that play a crucial role in regulating human development, growth, metabolism, and immune function. As the most important regulatory hormones in the stress response, glucocorticoids exhibit significant anti-inflammatory and immunosuppressive functions, and are widely used in clinical practice (Abtahi et al. 2022; Grodzielski et al. 2023). By inhibiting the activity of pro-inflammatory mediators such as TNF-α, interleukin-1 (IL-1), and IL-6, glucocorticoids decrease the infiltration of neutrophils and macrophages into the wound site, which is crucial in controlling acute inflammation and preventing the transition to a chronic wound state (Ricci et al. 2021). Studies have shown that following acute trauma, the massive secretion of glucocorticoids can enhance survival by improving the viability of trauma-induced myeloid-derived suppressor cells (TIMDSCs) (Zhang et al. 2012). Additionally, glucocorticoids are used to reduce graft rejection after lens and corneal transplants, possibly by promoting epithelial remodeling of postoperative wounds through the regulation of osteoprotegerin (OPG), brain-derived neurotrophic factor (BDNF), epiregulin (EREG), and nerve growth factor (NGF) expression (Kadmiel et al. 2016).

However, despite their anti-inflammatory properties, long-term local use of glucocorticoids has relatively negative effects on the skin. Research has found that prolonged use of corticosteroids significantly reduces the recovery rate of skin barrier function, leading to increased trans-epidermal water loss, which can be as much as three times the normal level (Niculet et al. 2020). This increased water loss not only affects the skin’s moisturizing capability but can also result in drier skin. After 3–4 weeks of topical treatment, the lipid content in the stratum corneum may decrease by more than 24%, further impacting skin health and its ability to retain moisture (Røpke et al. 2017; Niculet et al. 2020). Long-term application of glucocorticoids may also lead to atrophy of the epidermis and dermis, resulting in overall thinning of the skin. This thinning may be accompanied by changes in skin texture, telangiectasia, and purpura, which are especially noticeable in high-absorption areas such as the face, neck, axillae, perineum, and genitalia, and are particularly common in the elderly (Tanei 2020). Moreover, glucocorticoids can impair skin barrier function and delay the recovery of epidermal function (Scherholz et al. 2019), and prolonged use may cause dryness, scaling, erythema, and allergic reactions, and the recovery from these symptoms can take a considerable amount of time (Niculet et al. 2020). In addition to the direct effects on the skin, systemic use of glucocorticoids can also cause a range of issues, such as increased risk of infection, osteoporosis, and muscle atrophy, although these effects do not directly impact the skin (Lee et al. 2020). Therefore, when considering the use of glucocorticoids, it is crucial to carefully weigh their therapeutic benefits against the potential side effects. Thus, glucocorticoids should be used cautiously under medical supervision to ensure the safety and efficacy of the treatment.

## Summary and future perspective

**Fig. 2:**
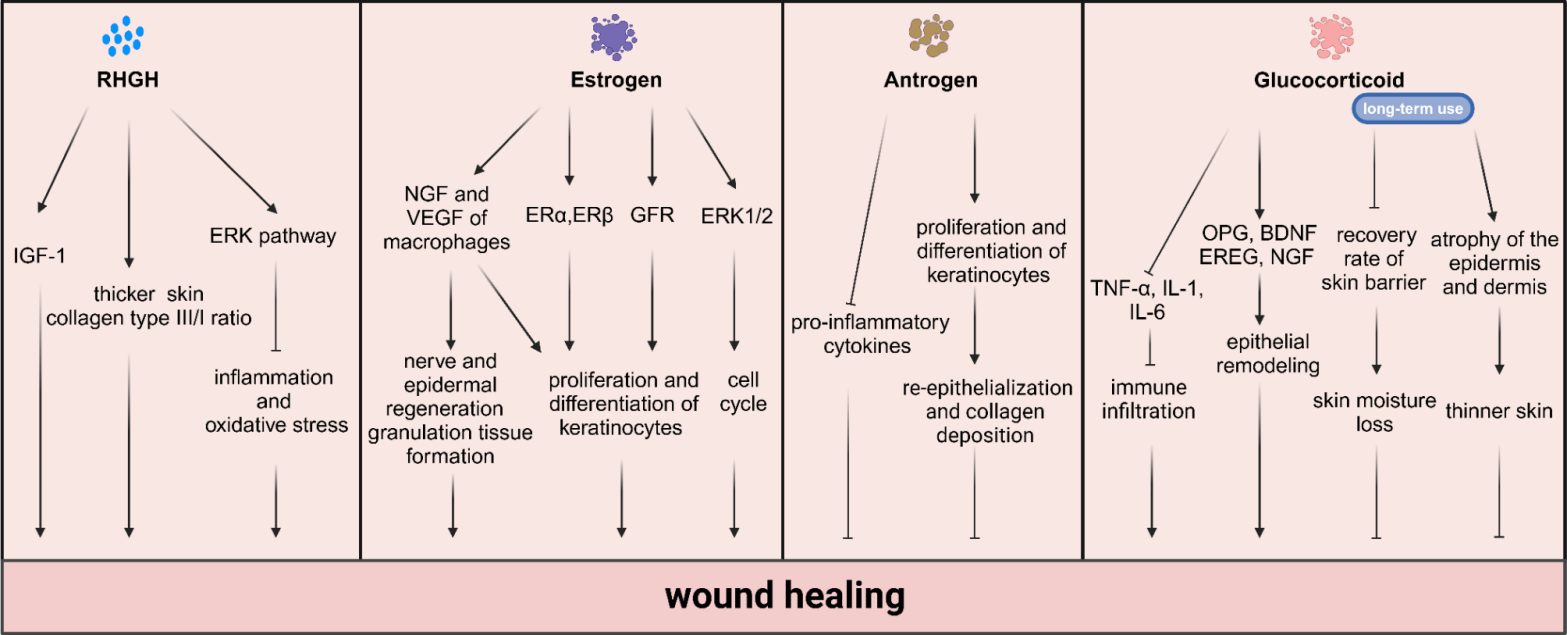
Distinct hormones and signaling pathways during wound healing. Highlights the importance of multiple cytokines, growth factors, and signaling pathways in orchestrating the wound healing cascade

Hormone therapy exhibited broad clinical application prospects (Akishita 2009), but also faced several challenges and limitations. Clinically, to maximize therapeutic effects and minimize side effects, precise control over the dosage and administration of hormone therapy is necessary. This may involve controlling the drug release rate, selecting appropriate administration routes, and planning treatment cycles. Furthermore, patient-specific factors such as age, gender, health status, and type of wound could influence the effectiveness of hormone therapy. Therefore, personalized treatment plans needed to be formulated based on the individual patient’s condition. Nevertheless, there might be synergistic or antagonistic interactions between different hormones (Zhou et al. 2022), which could affect therapeutic outcomes. In-depth research into these interactions would help optimize treatment strategies and achieve more effective therapies. Most importantly, the long-term effects of hormone therapy were not yet fully understood, necessitating continuous clinical follow-ups to evaluate its safety. As the mechanisms by which hormones contribute to wound healing are complex and diverse (Fig. 2), a thorough understanding of these mechanisms is crucial for developing new treatment strategies and mitigating the side effects of the current treatment regimen.

Future research should focus on the molecular mechanisms of hormone action, clinical outcome assessments of hormone therapy, and the development of individualized treatment plans. Through these studies, more effective and safer treatment options could be provided for trauma patients, thereby enhancing the overall efficacy and reducing the side effects of hormone therapy.

## Data Availability

No datasets were generated or analysed during the current study.

## Abbreviations

BDNF: Brain-derived neurotrophic factor
DHT: Dihydrotestosterone
EGF: Epidermal growth factor
Erα: Estrogen receptor α
Erβ: Estrogen receptor β
EREG: Epiregulin
ERK: Extracellular signal-regulated kinase
IGF-1: Insulin-like growth factor-1
IL-1: Interleukin-1
IL-6: Interleukin-6
NGF: Nerve growth factor
OPG: Osteoprotegerin
PCL: Polycaprolactone
REGF: Recombinant human epidermal growth factor
RHGH: Recombinant human growth hormone
SHBG: Sex hormone-binding globulin
TGF-β1: Transforming growth factor beta 1
TNF-α: Tumor necrosis factor-α
VEGF: Vascular endothelial growth factor
